# Editorial: The prevention and treatment of diabetic osteoporosis

**DOI:** 10.3389/fendo.2025.1629983

**Published:** 2025-06-02

**Authors:** Bingzi Dong, Kexin Zhang, Chunping Zeng

**Affiliations:** ^1^ Department of Endocrinology and Metabolism, The Affiliated Hospital of Qingdao University, Qingdao, China; ^2^ Department of Endocrinology and Metabolism, Clinical Research Center, Shandong Provincial Key Medical and Health Discipline of Endocrinology, Affiliated Hospital of Shandong Second Medical University, Weifang, China; ^3^ Department of Endocrinology, The Fifth Affiliated Hospital of Guangzhou Medical University, Guangzhou, China

**Keywords:** diabetes mellitus, osteoporosis, diabetic osteoporosis, prevention, therapy

Diabetes mellitus (DM) and osteoporosis represent major metabolic disorders with significant morbidity (1–3). Diabetic osteoporosis (DOP) is a diabetes-related complication and a metabolic bone disorder characterized by impaired bone microarchitecture, increased bone fragility, and significantly increased risk of bone fractures even with less reduced bone mass (4, 5). Compared to individuals without DM, those with DM have a significantly higher incidence of osteoporosis and related fractures (6). DOP has become a prominent clinical challenge, further complicated by factors such as poor glycemic control, mineral deficiencies, diabetes-related vascular and neuropathy complications, and the adverse effects of certain anti-diabetic medications. Traditional tools for assessing bone density and fracture risk often underestimate the actual risk in DM patients, underscoring the need for improved diagnostic methods. In terms of treatment, despite promising options such as glucagon-like peptide-1 analogues showing both glucose-lowing effect and bone anabolic benefit for diabetic osteoporosis (DOP), significant challenges remain, and further research is needed to optimize efficacy and safety (7–9). In this Research Topic, seven studies, including five original research articles and two review articles, provide new insights into the pathogenesis, biomarkers, and potential therapeutic approaches, offering promising directions for future research and clinical management.

Emerging evidence points to extracellular vesicles (EVs) as crucial regulators of the bone microenvironment in diabetes. In a comprehensive review, Jia et al. highlighted the role of EVs in mediating intercellular communication between osteoblasts and other bone-related cells. EVs were shown to transfer miRNAs and proteins that mitigate inflammation, ferroptosis, and the accumulation of advanced glycation end products, while promoting angiogenesis and osteogenesis. Importantly, the review also emphasized translational challenges, such as the scalable production and targeted delivery of EVs, which must be addressed to realize their therapeutic potential in bone remodeling under diabetic conditions.

Advancements in biomarker research further deepen our understanding of bone health in patients with type 2 diabetes (T2DM). Xu et al. utilized NHANES data to evaluate the advanced lung cancer inflammation index (ALI), a composite indicator derived from BMI, serum albumin, and neutrophil-to-lymphocyte ratio. Their findings revealed that lower ALI scores were associated with a higher prevalence of osteoporosis and reduced femoral bone mineral density (BMD), suggesting ALI may serve as a convenient, cost-effective screening tool in routine clinical practice. Similarly, Zhao et al. explored the relationship between serum insulin-like growth factor-1 (IGF-1) and BMD in T2DM patients. They identified a nonlinear association to show that IGF-1 SDS levels were positively correlated with BMD at multiple skeletal sites. These studies collectively highlight the importance of endocrine markers in early risk stratification and management of osteoporosis in DM population.

To probe the underlying mechanisms linking T2DM to osteoporosis, Yang et al. adopted a Mendelian randomization approach to investigate whether metabolic traits mediate the causal link between T2DM and osteoporosis. Despite confirming a robust causal relationship, none of the 30 glycemic, lipid, amino acid, or inflammatory markers examined were found to mediate this link. It suggests that the pathogenic mechanisms beyond systemic metabolic dysregulation, possibly includes bone-specific cellular dysfunction or gene–environment interactions.

The clinical burden of diabetes extends beyond bone fragility to encompass quality of life. In a population-based study, Li et al. evaluated health-related quality of life (HRQoL) among individuals with normal glucose levels, prediabetes, and diabetes.

This study found that both prediabetes and diabetes are associated with reduced health-related quality of life (HRQoL) compared to individuals with normal glycemic levels. The negative impact on HRQoL was especially pronounced among women, older adults, and individuals with obesity. These findings underscore the need for comprehensive, patient-centered strategies that not only prevent fractures but also address broader well-being in at-risk populations.

From a therapeutic standpoint, Jeddi et al. reviewed the therapeutic potential of nitrate, a nitric oxide donor, in DOP management. Acting through the nitric oxide/cGMP/PKG signaling pathway, nitrate has demonstrated efficacy in promoting bone formation, inhibiting resorption, and improving insulin sensitivity. Its dual benefits on bone and glucose metabolism present a compelling case for further exploration as a pharmacological strategy in diabetic bone disease. However, as the review noted, further clinical trials are required to validate its long-term safety and efficacy in diverse diabetic populations.

In terms of diagnostic innovation, Dehnen et al. evaluated the effectiveness of Cortical Backscatter (CortBS) ultrasound in assessing bone quality and fracture risk in patients with diabetes. Compared to conventional dual-energy X-ray absorptiometry, CortBS more accurately detected diabetes-related microstructural changes in cortical bone and demonstrated superior performance in discriminating fracture risk.

Together, these articles present a multifaceted view of DOP, spanning pathophysiological insights, biomarker discovery, therapeutic innovation, and diagnostic refinement. They collectively emphasize the importance of moving beyond traditional bone density assessments to incorporate inflammatory, endocrine, and structural evaluations tailored to the diabetic context. Moreover, they highlight the need for personalized and interdisciplinary approaches to improve the prevention, diagnosis, and treatment of this complex and increasingly prevalent condition. Further studies are warranted to validate these findings, refine diagnostic tools, and translate emerging therapies into clinical practice.
